# Additional effects of acupuncture on early comprehensive rehabilitation in patients with mild to moderate acute ischemic stroke: a multicenter randomized controlled trial

**DOI:** 10.1186/s12906-016-1193-y

**Published:** 2016-07-18

**Authors:** Lifang Chen, Jianqiao Fang, Ruijie Ma, Xudong Gu, Lina Chen, Jianhua Li, Shouyu Xu

**Affiliations:** Department of Acupuncture, The Third Affiliated Hospital of Zhejiang Chinese Medical University, 219 Moganshan Road, Xihu District, Hangzhou City, Zhejiang Province 310005 China; The Third Clinical Medical College, Zhejiang Chinese Medical University, 548 Binwen Road, Binjiang District, Hangzhou City, Zhejiang Province 310053 China; Department of Rehabilitation, The Second Hospital of Jiaxing, 1518 North Huancheng Road, Jiaxing, Zhejiang Province 314000 China; Department of Rehabilitation, Hangzhou First People’s Hospital, 261 Huansha Road, Hangzhou, Zhejiang Province 310006 China; Department of Rehabilitation, Sir Run Run Shaw Hospital College of Medicine Zhejiang University, No. 3 East Qingchun Road, Hangzhou, Zhejiang Province 310016 China

**Keywords:** Stroke, Acupuncture, Rehabilitation, Randomized controlled trial

## Abstract

**Background:**

Acupuncture is not considered a conventional therapy for post-stroke sequelae but it might have some additional positive effects on early rehabilitation. We conducted this trial to determine whether acupuncture has additional effects in early comprehensive rehabilitation for acute ischemic stroke and dysfunctions secondary to stroke.

**Methods:**

Two hundred fifty patients were randomized into two groups: acupuncture (AG) or no acupuncture (NAG). Eighteen acupuncture treatment sessions were performed over a 3-week period. The primary outcome was blindly measured with National Institutes of Health Stroke Scale (NIHSS) at week 1, week 3, and week 7. Secondary outcomes included: Fugl-Meyer Assessment (FMA) for motor function, bedside swallowing assessment (BSA) and videofluoroscopic swallowing study (VFSS) for swallowing function, the Mini-Mental State Examination (MMSE) and Montreal Cognitive Assessment (MoCA) for cognitive function, and the adverse reaction of acupuncture for safety assessment.

**Results:**

Significant improvements from acupuncture treatment were observed in NIHSS (*p* < 0.001), VFSS (*p* < 0.001), MMSE (*p* < 0.001), MoCA (*p* = 0.001), but not obtained from FMA (*p* = 0.228). Changes from baseline of all above variables (except FMA) also had the same favorable results. A significant improvement in FMA lower extremity subscale appeared in AG (*p* = 0.020), but no significant difference was found for the upper extremity subscale (*p* = 0.707). More patients with swallowing disorder recovered in AG (*p* = 0.037). Low incidence of mild reaction of acupuncture indicated its safety.

**Conclusions:**

This trial showed acupuncture is safe and has additional multi-effect in improving neurologic deficits, swallowing disorder, cognitive impairment, and lower extremity function, but has no significant improvement for upper extremity function during this short-term study period.

**Trial registration:**

Chictr.org ChiCTR-TRC −12001971 (March 2012).

**Electronic supplementary material:**

The online version of this article (doi:10.1186/s12906-016-1193-y) contains supplementary material, which is available to authorized users.

## Background

In China, there are over seven million stroke survivors (65 % ischemic stroke), only 1.3 % or fewer received IV rt-PA in the effective time window [1, 2]. Stroke patients who receive early comprehensive rehabilitation in a stroke unit are more likely to be alive, independent and have better functional outcomes [3]. Acupuncture (includes electroacupuncture and scalp acupuncture) is well accepted in China for poststroke rehabilitation as part of their comprehensive rehabilitation strategies.

To date the question about whether acupuncture should be used as a routine treatment for stroke and stroke-related dysfunctions is a hot and controversial topic. Some studies showed positive but limited effectiveness of acupuncture as an adjunct treatment to the conventional rehabilitation [4–6]. On the other hand, some research showed acupuncture had no beneficial effect for functional recovery, activities of daily living (ADL) and health-related quality of life (QOL) after stroke [7–9]. The potential additional positive effects of acupuncture for stroke should be confirmed by further high-quality clinical studies with greater statistical power [10–14]. We conducted a multicenter randomized controlled trial to demonstrate whether acupuncture could additionally improve neurological deficit (main aim), motor function, swallowing disorder, and cognitive impairment (secondary aims) during the acute ischemic stroke with a multi-effect.

## Methods

### Subjects

#### Inclusion criteria

Patients between 35–80 years of age hospitalized with acute ischemic stroke and hemiplegia were included. Patients with onset of stroke between two to seven days beforehand, the NIHSS score between 5 and 14, and displaying clear consciousness and stable vital signs were only included if it was their first incidence of stroke. We excluded severe stroke (the NIHSS score between 15 and 24) after the trial commencement, because the severe stroke patients need more frequently skilled care, which may be beyond our treatment program.

### Exclusion criteria

Excluded from the study were patients who suffered from serious heart, liver, and kidney-related diseases, or blood coagulation dysfunction, and patients unable to complete the MMSE test or bedside swallowing assessment (BSA). Congenital disabilities were also excluded. Patients who suffered POCI (Posterior Circulation Infarcts) in OCSP (Oxfordshire Community Stroke Project) classification, or received thrombolytic therapy or who participated in other clinical trials within previous three months, and women who were pregnant or breast-feeding were also excluded from this study.

### Randomization and blinding

Consecutive patients were randomly assigned to standard rehabilitation care with or without acupuncture (1:1 allocation ratio). Randomization was computer-generated by independent research staff using software, and the generated list of random numbers was placed into sequentially numbered, opaque, sealed envelopes. Study coordinators who trained before the trial and did not participate in treatment or nursing, informed the eligible subjects whether they would receive acupuncture treatment or not. The specialized acupuncturists were informed to do acupuncture for designed patients. All of the allopathic medical staff, rehabilitation therapists, outcome assessors, and data analysts were blinded to group assignments.

### Interventions and comparison

Both groups received conventional stroke rehabilitation care according to Chinese stroke rehabilitation treatment guidelines [15], which began as soon as the diagnosis of stroke was established and life-threatening conditions were under control. The basic conventional rehabilitation care included normal limb posture, passive motion with hemiplegia side, bedside rehabilitation (Bobath technique, overturning movement, bridge movement), neuromuscular electrical stimulation, and/or swallowing training for dysphagia, and/or cognitive training for cognitive impairment. The rehabilitation program (including physiotherapy and occupational therapy for two hours per day, six days per week) for each participant was developed by the rehabilitation team according to the investigator’s brochure. The acupuncture group also received additional thirty minutes of acupuncture therapy as bedside treatment, six days per week for three weeks (eighteen total sessions). Scalp needles were retained for four hours (specific acupuncture treatment details outlined in ‘Manipulations’ section). Patients in the control group only received conventional stroke rehabilitation care, which was the same as the acupuncture group. However, no acupuncture treatments were given during the whole study period to the control group.

#### Acupuncture points

The acupuncture was performed by three acupuncture doctors who have a master degree with more than five years of clinical experience, and had been trained previously to perform the same protocols. For scalp acupuncture, two to three needles were penetrated through the top midline, the motor region (MS-6), and the sensory region (MS-7) of the lesion side. The points for the affected side of the body acupuncture were as follows: LI15 (Jianyu), LI11 (Quchi), LI10 (Shousanli), TE5 (Waiguan), and LI4 (Hegu) for upper extremities; ST34 (Liangqiu), ST36 (Zusanli), GB34 (Yanglingquan), SP6 (Sanyinjiao), ST40 (Fenglong), ST41 (Jiexi), and LR3 (Taichong) for lower limbs. The points for dysphagia were added as follows (this group of acupoints was named “nape-acupuncture”): GB20 (Fengchi), EX-HN14 (Yiming), BL10 (Tianzhu), GV16 (Fengfu), Gongxue (1 cun below GB20), and CV23 (Lianquan). For cognitive impairment, GV20 (Baihui), GV24 (Shenting), GB13 (Benshen), EX-HN-1 (Sishencong) were added (The location of acupoints was detailed in Additional file 1: Table S1).

#### Manipulations

Manipulations of scalp acupuncture: the needles were swiftly inserted into the subcutaneous tissue of the scalp in a horizontal direction. When acupuncture needles (the stainless steel needle, 0.25 mm × 40 mm in size, Huatuo brand, manufactured by Suzhou Medical Appliance in Jiangsu Province of China) were reached the subgaleal layer and the practitioner felt the weak insertion resistance, needles were further inserted to the depth of 30 mm ~ 40 mm by twirling method. Patients were placed in a sitting position during the needle insertion, but they could move freely during the four-hour retention. Manipulations of body acupuncture: the needle was inserted into the points to a depth of between 30 mm and 40 mm according to different regions in a supine position. Manual stimulation was applied to the body acupoints until the patients experienced the needling sensation (called Deqi in Chinese acupuncture) [16]. For electroacupuncture of LI15 (Jianyu) and LI11 (Quchi), ST36 (Zusanli) and SP6 (Sanyinjiao) points, GB6805-2 Electro & Acu-point Stimulators (Huayi Medical Supply & Equipment Co. Ltd, Shanghai, China) were used to give intermittent wave and low-frequency (two Hz) stimulus. The intensity was within the scope that patients could tolerate. For nape-acupuncture on dysphagia: after the scalp acupuncture needle insertion, patients with dysphagia received nape-acupuncture in a sitting position during the needle insertion, then lied supine with a pillow padded under the occiput to avoid the needles touching the bed. The shorter size of 0.25 mm × 25 mm needles were used for nape-acupuncture for safety consideration. The retention of body acupuncture and nape-acupuncture was thirty minutes. The order of acupuncture treatment was as follows: For insertion: scalp acupuncture-nape-acupuncture (for dysphagia)-body acupuncture and electro-acupuncture; For withdrawing needles: the electro-acupuncture-body acupuncture-nape-acupuncture-the scalp acupuncture. Minimal interaction with the provider was necessary, as needles were inserted in 5–7 min, the patient was left alone to rest for thirty minutes, with no additional attention, mobility/posture training, or interaction. Needles were removed quickly, within 1–2 min, and social interaction during the acupuncture session was minimal.

### Outcome measurements

Participants received rehabilitation assessments at week 0 (baseline), week 1, week 3 (after treatment), and week 7 (follow-up). The NIHSS index for neurologic deficit evaluation was used as the primary outcome measurements. Secondary outcomes included the FMA scale for motor functions, the rate of recovery based on the bedside swallowing assessment (BSA) and the videofluoroscopic swallowing study (VFSS) for swallowing function, the mini-mental state examination (MMSE) and Montreal cognitive assessment (MoCA) for cognitive function, and adverse reaction of acupuncture for safety evaluation. We changed the measurement of swallowing function before the very commencement of the clinical trial. Instead of using “Depression cropland of swallowing ability evaluation”, the outcome measure was switched to BSA and VFSS to more effectively and accurately evaluate the swallowing function.

### Statistical analysis

Sample size was calculated based on the results of our preliminary test and previous studies, planned separate analysis with the change in NIHSS scores, which was detailed in our study protocol [17]. Analysis was made by blinded biostatisticians using the SPSS software (version 17.0), used two-sided significance tests at the 5 % significance level. Efficacy and safety analysis were conducted according to the intention-to-treat (ITT) principle. The full analysis set (FAS) included all randomized patients who met the major eligibility criteria, had received at least one acupuncture treatment, and had undergone at least one assessment for all of the measurements. The per protocol set (PPS) was conducted in the sensitivity analysis. The analysis of swallowing disorder and cognitive impairment were performed only for the population who tested positive at baseline (Table 1, legend a). Missing values were imputed by the last-observation-carried-forward method. Continuous variables in this trial were all normally distributed and expressed as mean (SD). Independent sample *t*-tests were used to compare the difference between two groups; Repeated measures ANOVA was used to predict values of measurements across time. Categorical variables were expressed as counts and percentages.

**Table 1:** Baseline characteristics of trial participants

Variable	AG (*N* = 125)^a^	NAG (*N* = 125)^a^	*P*
Age, Mean (SD), y	62.52(10.60)	64.06(10.54)	0.231
Male sex, n (%) History of stroke, Mean (SD) d	74(59.20) 4.53(1.13)	74(59.20) 4.48(1.32)	1.000 0.738
Left side of hemiparesis, n (%)	83(66.40)	79(63.20)	0.596
OCSP infarct classification, n (%)			0.847
TACI	22(17.60)	19(15.20)	
PACI	70(56.00)	71(56.80)	
LACI	25(20.00)	29(23.20)	
Unknown/Other	8(6.40)	6(4.80)	
NIHSS, Mean (SD)	8.31(2.43)	7.75(2.72)	0.054
FMA, Mean (SD)	46.06(14.96)	49.28(15.90)	0.175
BSA positive, n (%)^a^	103(82.40)	97(77.60)	0.343
VFSS Assessment, n (%)^b^	68(66.02)	65(67.01)	0.881
VFSS, Mean (SD)^b^	5.26(1.57)	5.55(1.76)	0.319
Cognitive impairment, n (%)^a^	78(62.40)	71(56.80)	0.367
Education level, (years)^c^			0.397
0–5, n (%)	20(25.64 %)	21(29.58 %)	
5–8, n (%)	29(37.18 %)	31(43.66 %)	
>8, n (%)	29(37.18 %)	19(26.76 %)	
Age, mean (SD), y^c^	63.85(10.61)	64.06(10.39)	0.903
MoCA, Mean (SD)	18.27(2.01)	18.35(2.11)	0.806
MMSE, Mean (SD)	20.86(2.64)	20.97(2.65)	0.795

Abbreviations: *AG* Acupuncture group, *NAG* No acupuncture group, *OCSP* Oxfordshire Community Stroke Project, *TACI* Total anterior circulation infarcts, *PACI* Partial anterior circulation infarcts, *LACI* Lacunar infarcts, *NIHSS* National Institutes of Health Stroke Scale, *FMA* Fugl-Meyer Assessment, *BSA* Bedside Swallowing Assessment, *VFSS* Video-fluoroscopic Swallowing Study, *MoCA* Montreal Cognitive Assessment, *MMSE* Mini-mental State Examination

^a^FAS analysis included 250 cases. The analysis of swallowing disorder was made on the cases who tested positive for the BSA, the analysis of cognitive impairment was made on the cases who tested MMSE ≤ 24 and MoCA ≤ 20

^b^Of 200 swallow disorders, 133 patients received VFSS assessment voluntarily, others could not participate due to physical limitations or refused to accept the test

^c^Patients with cognitive impairment should be first compared with education level and age. 0–5 years education level means elementary school, 5–8 years education level means middle school, >8 years education level means above high middle school

Baseline data was collected and compared first by independent sample *t*-test and χ^2^ test. Values of NIHSS, FMA, VFSS, MoCA, and MMSE were compared by repeated measures ANOVA across two to four testing timepoints (week 0, week 1, week 3, and week 7). Differences between two groups of all outcome value changes (week7-week0) were compared by independent sample *t*-test. FMA motor subscales of upper extremities (UE) and lower extremities (LE) were calculated and analyzed by *t*-test. The recovery rate of swallowing function based on BSA was compared by *χ*^2^ test. Acupuncture-related safety analysis was performed by analyzing the frequency of adverse events suspected as related to acupuncture treatment.

### Ethical standard, registrations, patient consents, and data management

Each ethics committee of the Third Affiliated Hospital of Zhejiang Chinese Medical University, The Second Hospital of Jiaxing City, Hangzhou First People’s Hospital, and together with Sir Run Run Shaw Hospital College of Medicine Zhejiang University, all approved the study (Additional file 2). All patients gave their written informed consent before participating in the study. A Data and Safety Monitoring Board reviewed the performance and safety of the trial monthly. The study was conducted in accordance with the Declaration of Helsinki, registered with Chictr.org (ChiCTR-TRC-12001971), and reported according to CONSORT 2010 guidelines (Additional file 3). A detailed study protocol has previously been published [17] (Additional file 4).

The Case Report Form (CRF), Treatment Form and Adverse Events Form were first completed on paper copies and then double entered into the Electronic Data Capture (EDC) System electronically by two independent investigators. The original CRFs and all other forms (including the consent forms) were archived securely at The Third Affiliated Hospital of Zhejiang Chinese Medical University for five years following publication of the last paper or report from the study.

## Results

### Participants

We recruited participants from two cities (Hangzhou and Jiaxing, China) via advertisements by local newspapers, health-related TV programs, the Internet, and posters in communities and hospitals between March 1, 2012 and June 30, 2014.

**Fig. 1:**
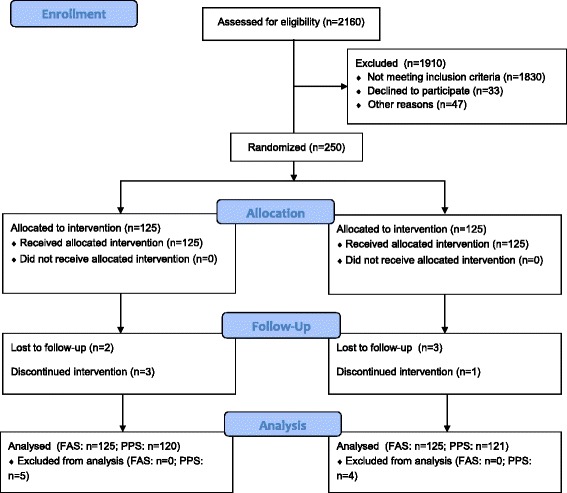
Flow of participants through the trial

During the study period, 2160 patients were assessed for eligibility, 1830 were not interested in participation or had obvious violation of inclusion criteria (Fig. 1). Of the 330 patients who entered at baseline, eighty were excluded because of various reasons. Among the eighty patients, 33 of them declined to participate, most of them insisted on receiving a comprehensive rehabilitation combined with Traditional Chinese Medicine (TCM) and western medicine; 47 of them were excluded because of other reasons, such as complication, poor compliance, early discharge, etc. Two hundred and fifty patients were strictly included in the study and were randomly assigned to the two treatment groups (AG or NAG). Three failed to complete treatment, and two were lost to follow-up in AG. One exited due to recurrent stroke, and three were lost to follow-up in NAG. There was zero mortality in our trial. One hundred twenty participants of AG and one hundred twenty-one participants of NAG completed all of the treatments and measurements and were included in PPS. One hundred twenty five subjects of each group were included in the FAS. We presented efficacy results in this paper only from the PPS, because the efficacy results of FAS and PPS were consistent. Table 1 shows fairly equal groups in terms of demographics and clinical characteristics. A large number of stroke patients admitted to the three hospitals in our study provided us a plentiful source of subjects with high quality and good compliance.

### Efficacy of primary and secondary outcomes

All variables at each testing time point are detailed in Table 2. NIHSS was estimated at week 0 (baseline), week 1 (during the intervention), week 3 (after treatment), and week 7 (follow-up); FMA, MMSE, and MoCA were assessed at week 0, week 3, and week 7; VFSS was evaluated at week 0 and week 7. No differences were found in all above variables between two groups at each testing time point.

**Table 2:** Variables at each testing time point, Mean (SD)

Variables	Group (N)	Week 0	Week 1	Week 3	Week 7
NIHSS	AG (120)	8.33(2.43)	6.61(2.47)	4.95(2.44)	3.48(2.26)
	NAG (121)	7.71(2.71)	6.20(2.69)	4.43(2.53)	3.66(2.25)
FMA	AG (120)	46.12(14.96)	–	54.21(14.70)	64.41(14.17)
	NAG (121)	50.25(15.90)	–	57.87(16.84)	66.85(16.51)
VFSS	AG (68)	5.26(1.57)	–	–	9.77(0.45)
	NAG (65)	5.55(1.76)	–	–	9.35(0.77)
MMSE	AG (78)	20.86(2.64)	–	22.51(2.67)	23.80(2.16)
	NAG (71)	20.97(2.64)	–	22.08(2.74)	23.12(2.95)
MoCA	AG (78)	18.27(2.01)	–	20.02(2.34)	21.51(2.26)
	NAG (71)	18.35(2.11)	–	19.56(2.36)	20.78(2.45)

No differences were found between two groups at each testing time point. NIHSS was estimated at week 0 (baseline), week 1 (during the intervention), week 3 (after treatment), and week 7 (follow-up); FMA, MMSE, and MoCA were assessed at week 0, week 3, and week 7; VFSS was evaluated at week 0 and week 7

For the primary outcome of neurologic deficit evaluated based on the change for NIHSS, significant interaction between time and group was observed in the repeated measures ANOVA (Interaction effect *F* = 9.33, *p* < 0.001) (Table 3). A bigger score change (week7-week0) of NIHSS due to acupuncture was also revealed by *t*-test result (*T* = −4.13, *p* < 0.001) (Table 4).

**Table 3:** Results of repeated measures ANOVA for outcomes (PPS)

Variables	*df*	*df*	Effects	F	*P*	Significance
	Effect	Error				
NIHSS	1	239	Group	1.72	0.192	NS
	3	237	Time	675.34	<0.001	S
	3	237	Group by time	10.69	<0.001	S
FMA	1	239	Group	2.83	0.094	NS
	2	238	Time	775.87	<0.001	S
	2	238	Group by time	2.04	0.133	NS
VFSS	1	131	Group	0.502	0.480	NS
	1	131	Time	1500.16	<0.001	S
	1	131	Group by time	11.50	0.001	S
MMSE	1	147	Group	0.62	0.433	NS
	2	146	Time	316.24	<0.001	S
	2	146	Group by time	8.74	<0.001	S
MoCA	1	147	Group	1.06	0.305	NS
	2	146	Time	350.57	<0.001	S
	2	146	Group by time	8.00	0.001	S

*df* indicates degrees of freedom. *NS*, not significant; *S*, significant

**Table 4:** Value changes (week 7-week 0) of variables by independent samples *t*-test (PPS)

Variable	Group (N)	week 7-week 0 Mean (SD)	95 % CI of the Difference	*T*	*P*
NIHSS	AG(120)	−4.85(1.63)	−1.22, −0.43	−4.13	<0.001
	NAG(121)	−4.05(1.52)			
FMA	AG(120)	18.29(7.32)	−0.77, 2.85	1.13	0.259
	NAG(121)	16.60(10.41)			
FMA (UE)	AG(120)	7.26(3.40)	−0.68, 1.00	0.14	0.707
	NAG(121)	7.10(3.22)			
FMA (LE)	AG(120)	11.83(3.72)	0.18, 2.01	5.52	0.020
	NAG(121)	10.73(3.54)			
VFSS	AG(68)	4.51(1.39)	0.27,1.16	3.17	0.002
	NAG(65)	3.80(1.20)			
MMSE	AG(78)	2.95(1.26)	0.39, 1.20	3.90	<0.001
	NAG(71)	2.15(1.23)			
MoCA	AG(78)	3.24(1.40)	0.38, 1.23	3.77	<0.001
	NAG(71)	2.44(1.19)			

FMA (UE) = FMA subscale of upper extremity (66 points); FMA (LE) = FMA subscale of lower extremity (34 points)

FMA, one of the secondary outcomes with a total possible score of 100 points, includes an assessment of the upper extremity (UE, 66 points) and lower extremity (LE, 34 points) [18]. When we analyzed the total FMA motor score, there was no significant difference, either with repeated measures ANOVA (Interaction effect *F* = 1.49, *p* = 0.228) (Table 3) or with *t-*test for value changes from baseline (*T* = 1.13, *p =* 0.259) (Table 4). However, the changes of subscale scores of UE and LE showed different results. The score change of UE was generally similar in two groups (*T* = 0.14, *p =* 0.707), but the change of LE was much bigger in AG (*T* = 5.52, *p* = 0.020) (Table 4).

Swallowing function is another secondary outcome we examined. One hundred three cases (87.4 %) in AG and ninety-seven cases (77.6 %) in NAG suffered swallowing disorder according to BSA, which was conducted by nurses with a teaspoon (5 ml) of water 3 times (stage 1) and 60 ml of water in a beaker (if the swallowing tested normal in stage one) [19]. We used a VFSS assessment scale according to videofluoroscopic dysphagia criteria [20] (Additional file 5: Table S2). The total score of 10 indicated normal. Of 200 swallowing disorders identified, 133 cases received VFSS examination, 68 in AG and 65 in NAG respectively. The baselines of the percentage of dysphagia and the score of VFSS findings in two groups were comparable (*p* = 0.343, *p* = 0.319) (Table 1). The repeated measures ANOVA revealed a significant interaction between time and group (Interaction effect *F* = 23.62, *p* = <0.001) (Table 3). The independent sample *t*-test demonstrated the change (week7-week0) of VFSS score of AG was significantly higher than that of NAG (*T* = 3.17, *p* = 0.002) (Table 4). The ratio of recovery was also significantly higher for the patients assigned acupuncture (*χ*^*2*^ *=* 4.35, *p* = 0.037) (Table 5).

**Table 5:** Recovery rate of swallow function between two groups

Time	Group (N)	N (%)	*χ* ^*2*^	*P*
3 weeks	AG(103)	48(46.60)	3.86	0.050
	NAG(97)	32(32.99)		
7 weeks	AG(103)	95(92.23)	4.35	0.037
	NAG(97)	80(82.47)		

Cognitive performance as measured by the MoCA and MMSE varies within the population by age and education [21]. We used the adjusted scores of MMSE and MoCA with new cutoffs (MMSE ≤ 24, MoCA ≤ 20) as the diagnostic criteria, which proved good sensitivity and very good specificity for both tests [22]. In our baseline period, 59.60 % patients overall suffered cognitive impairment. A significant interaction was found between the time and group with MMSE (Interaction effect *F* = 8.74, *p* < 0.001) and MoCA (Interaction effect *F* = 8.00, *p* = 0.001) (Table 3). The changes from baseline were also significantly bigger in AG with MMSE (*T* = 3.90, *p* < 0.001) and MoCA (*T* = 3.77, *p* = 0.001) (Table 4).

### Safety

To evaluate the safety of acupuncture, we summarized the adverse reaction of AG. Our study showed low incidence of mild symptoms with acupuncture for acute stroke (Additional file 6: Table S3). There were no acupuncture-related adverse events recorded (Additional file 7: Table S4).

## Discussion

Many reviews conclude acupuncture is potentially effective and safe in the treatment of acute ischemic stroke and sequelae, but further large, methodologically sound trials are still required to confirm or refute this [10, 23–30]. A recent large RCT included 862 subjects focused on death/disability to compare acupuncture plus standard care with standard care alone. Their trial demonstrated that fewer patients died or ended up dependent in the acupuncture group than in the control group [14]. Our trial treated patients with hemiplegia, dysphagia, and cognitive disorders after stroke simultaneously, and focused on specific and multiple effects of acupuncture in early comprehensive rehabilitation for acute ischemic stroke. Significant improvements of neurological deficit, lower extremity motor function, swallowing disorder, and cognitive impairment using acupuncture were demonstrated with strong evidence compared with conventional rehabilitation alone. As research shows, acupuncture may play effective roles in neuroprotective, microcirculatory, and metabolic recovery, which may accelerate the recovery of brain function in the early stage of stroke [11, 12, 31, 32]. Early and effective rehabilitation interventions can enhance the recovery process and minimize functional disability [33].

NIHSS, mainly developed for using in acute stroke trials, strongly predicts the likelihood of a patient’s recovery after stroke at early stage [34]. This measure was used as a primary outcome in this trial, and a good result of NIHSS may implicate a good functional recovery in a long-term process. Hemiplegia, dysphagia, and cognitive impairment are three common sequelae of acute stroke. When we recruited patients with stroke, most eligible participants were inevitably complicated with one or more than one dysfunction. These sequelae usually can be treated by acupuncture simultaneously through modification of acupoints. For example, we added GB20 (Fengchi), EX-HN-14 (Yiming), BL10 (Tianzhu), GV16 (Fengfu), Gongxue (1 cun below GB20), and CV23 (Lianquan) for swallowing disorder. This comprehensive treatment and appropriately tailored modification for stroke and sequelae can be considered one of the main advantages of acupuncture therapy. We focused on the potential application of acupuncture to treat not only poststroke neurological impairment, but also many common dysfunctions; and conducted precise and previously validated measurements to assess these outcomes. In this way, we may have conducted a more objective and comprehensive evaluation for the therapeutic effect of acupuncture on stroke rehabilitation. In order to provide stronger evidence of efficacy outcomes, we utilized MMSE and MoCA for assessing cognitive function and used VFSS, rate of recovery based on BSA for evaluating swallowing function. Different measurements on the same index obtained consistent good results for both cognitive and swallowing functions. Motor disorder is one of the most important items of the ability to carry out the ADL [35], and is also as apart of NIHSS (8 points of motor function assessment included in the total maximum 42 points). The different results of FMA total motor function and NIHSS implicate that acupuncture improves neurological deficit maybe through other aspects rather than motor function, such as perception [36].

The design of acupuncture treatment in this trial was flexible and integrated. Both scalp acupuncture and electroacupuncture were used for treating patients with acute stroke. These two techniques were both well developed and popularly used in modern society. The acupoints of body acupuncture were designed according to the classic traditional theory, and mainly distributed in the limbs of the affected side. Furthermore, we emphasized the feeling of “De Qi” for every patient who received acupuncture, which is considered to be essential and used as a predictor to the therapeutic effectiveness of acupuncture for stroke recovery [37]. However, improvement of limb motor function due to acupuncture was not observed with the FMA motor function index. Several previous trials of acupuncture on limb rehabilitation had the same negative result [38, 39].

These findings addressed a question which deserves pondering: Despite performing acupuncture treatment primarily on distal points mainly located in the extremities, why was the effect of acupuncture on limb function not as significant as the effect of acupuncture on neurological deficit, swallowing disorder and cognitive impairment? To partly explain this question, two aspects should be considered. First, one purpose of this study was to observe the effect of acupuncture for swallowing disorder, therefore, there was a screening-referral bias during enrollment. As a result, the incidence of swallowing disorder after acute stroke in this trial seemed to be much higher (80.0 %) than usual (47.0 %) [19]. There were 103 (82.40 %) participants in AG screened for swallowing disorder and received ten more acupoints on the neck area in addition to scalp acupuncture and body acupuncture. This “nape-acupuncture” may play a good role, not only for dysphagia but also for the recovery of neurological function. The mechanism of “nape-acupuncture” deserves further study. In addition to “nape-acupuncture”, the additional acupoints for cognitive impairment and the role of scalp acupuncture on stroke recovery cannot be ignored. Second, according to the concept of “holism” in TCM [40], acupoints in limbs especially those located below elbow and knee joints are very important in dealing with organ and meridian diseases. The therapeutic function of these points is not only for local problems, but also for the whole body.

An important finding in this clinical trial warrants additional discussion. The analysis of FMA subscale of UE and LE revealed very different results of acupuncture for upper extremity and lower extremity motor function. Acupuncture significantly improved LE rehabilitation but had no significant improvement on UE motor function during the 7-week study period. Maybe UE motor function is more difficult to be improved by acupuncture. A patient with stroke may be able to walk independently with limited motor recovery of LE, however, functional use of UE requires finer motor control and therefore a higher level of recovery [41–43]. Meanwhile, ADL measurement tools include a greater percentage of questions that focus on upper extremity tasks, therefore, UE hemiparesis has a significant impact on ADL measurements [39]. As we observed, studies using ADL or QOL to measure the comprehensive ability of patient with stroke were more likely to result in negative or contradictory outcomes for functional recovery after stroke [7, 8, 39, 44–47]. Our finding may provide a rationale for the mixed results in the studies mentioned above.

This trial also has limitations: 1) Only part of the patients with swallowing disorders received VFSS at baseline and 3 weeks after treatment, because some participates’ had to be excluded based on their physical conditions. 2) The study period in our study was short because of ethical and clinical reality. Many patients with stroke in China would choose acupuncture therapy in subacute stage (about one month after onset) to promote rehabilitation. We set follow-up as one month, so as not to restrict study participants from selecting comprehensive therapy in subacute stroke phase. The short study period may also be one of the reasons acupuncture treatment did not show a significant impact for upper extremity function. 3) We did not use sham acupuncture in the control group. Without a rigorous control for placebo effect, the results of the study may include a small placebo bias. However, many Chinese patients have previous experience with acupuncture or know it, making them able to easily distinguish a real acupuncture treatment from a sham control. These realities indicate the difficulty in conducting a placebo-controlled sham acupuncture trial in China. We paid more attention to reducing the bias by minimal interaction during treatments in the AG group and communication among participants of the two groups.

## Conclusions

In summary, although a large number of randomized controlled trials on acupuncture stroke treatment have been implemented over the past twenty years, most of these trials were dedicated to reveal the impact of acupuncture only for one particular dysfunction after stroke, such as hemiplegia, dysphagia, cognitive impairment, depression, as examples. Other RCTs focused on the effect of acupuncture for ADL, QOL, or mortality and disability, and most of these trials obtained negative results. Actually, patients with acute stroke not only suffered motor disorders, but also experienced swallowing disorders, cognitive impairment, or other dysfunctions at the same time. Conducting acupuncture treatment for patients with stroke, may take into account all dysfunctions, which can be treated with modification of acupoints. Since acupuncture may have potential effects for many sequelae after stroke, why not use it to treat these disorders at the same time? Acupuncture treatment towards multiple goals may produce better and more comprehensive results. This trial designed and evaluated acupuncture for multiple dysfunctions after stroke, and showed acupuncture has additional multi-effect at the early stage of ischemic stroke with strong evidences.

## Abbreviations

AG, acupuncture group; BSA, bedside swallowing assessment; FMA, fugl-meyer assessment; MMSE, mini-mental state examination; MoCA, montreal cognitive assessment; NAG, no acupuncture group; NIHSS, national institutes of health stroke scale; VFSS, video-fluoroscopic swallowing study

## Additional files

Additional file 1: Table S1. The location of acupoints. (DOCX 24 kb) Additional file 2: Ethical approvals of the ethical committees. (PDF 770 kb) Additional file 3: CONSORT checklist. (PDF 46 kb) Additional file 4: Study protocol: Acupuncture for acute stroke: study protocol for a multicenter, randomized, controlled trial. (PDF 369 kb) Additional file 5: Table S2. VFSS scoring criteria. (DOCX 20 kb) Additional file 6: Table S3. Summary of adverse reaction of acupuncture. (DOCX 19 kb) Additional file 7: Table S4. Incidence of adverse events. (DOCX 21 kb)
