# Supplementary material for: Additional effects of acupuncture on early comprehensive rehabilitation in patients with mild to moderate acute ischemic stroke: a multicenter randomized controlled trial
Source: BMC Complement Altern Med. 2016 Jul 18;16:226. doi: 10.1186/s12906-016-1193-y (PMC4950630; doi:10.1186/s12906-016-1193-y)
Supplement: Additional file 1: Table S1. — The location of acupoints. (DOCX 24 kb) [file 12906_2016_1193_MOESM1_ESM.docx]

Additional file 1: Table S1. The location of acupoints.

| Points | Location |
| --- | --- |
| LI15  (Jian yu) | In the depression distal and anterior to the acromion, between the clavicular and acromial portions of the deltoid muscle. |
| LI11  (Qu chi) | With the elbow flexed, on the lateral end of the elbow crease, in a depression between the end of the crease and the lateral epicondyle of the humerus, on the extensor carpi radialis longus muscle. |
| LI10  (Shou san li) | 2 cun distal to LI11, on the extensor carpi radialis longus muscle. |
| TE5  (Wai guan) | 2 cun proximal to the dorsal wrist joint space (dorsal wrist crease), between the radius and the ulna. |
| LI4  (He gu) | On the radial aspect of the hand, between the 1st and 2nd metacarpal bones, closer to the 2nd metacarpal bone and approximately at its midpoint. |
| ST34  (Liang qiu) | On a line joining the lateral patellar border and the anterior superior iliac spine, 2 cun proximal to the upper lateral border of the patella, in a groove of the vastus lateralis muscle. |
| ST36  (Zu san li) | 3 cun distal to ST-35 (‘lateral eye of the knee’) and 1 finger-breadth lateral to the anterior crest of the tibia, on the tibialis anterior muscle. |
| GB34  (Yang ling quan) | In the depression anterior and inferior to the head of the fibula, between the peroneus longus and extensor digitorum longus muscles. |
| ST40  (Feng long) | At the midpoint of the line joining ST-35 and ST-41, 2 fingerbreadths lateral to the anterior crest of the tibia. |
| SP6  (San yin jiao) | 3 cun proximal to the highest prominence of the medial malleolus, on the posterior border of the medial crest of the tibia. |
| ST41  (Jie xi) | On the ankle, on the level of the highest prominence of the lateral malleolus, in the depression between the tendons of the extensor digitorum and the extensor hallucis longus. |
| LR3  (Tai chong) | On the dorsum of the foot, between the 1st and 2nd metatarsal bones, in the depression proximal to the metatarsophalangeal joints and the proximal angle between the two bones. |
| GB20  (Fengchi) | At the lower border of the occipital bone, in the depression between the origins of the sternocleidomastoid and trapezius muscles. |
| Ex-HN-14  (Yi ming) | At the junction of the head and the occiput, posterior to the mastoid bone. |
| BL10  (Tian zhu) | Approximately 1.3 cun lateral to ➞ GV-15, where the trapezius muscle inserts on the lower border of the occiput, close to where the major occipital nerve emerges. |
| GV16  (Feng fu) | On the posterior midline, directly below the external occipital protuberance, in the depression between the origins of the trapezius muscle. |
| Gong Xue | 1.5 cun below GB20 |
| CV23  (Lian quan) | On the anterior midline, superior to the upper border of the hyoid bone. |
| GV20  (Bai hui) | At the junction of a line connecting the apices of the ears and the midline, 5 cun from the anterior or 7 cun from the posterior hairline respectively. |
| GV24  (Shen ting) | On the midline, 0.5 cun superior to the anterior hairline or 4.5 cun anterior to ➞ GV-20. |
| GB13  (Ben shen) | 3 cun lateral to ➞ GV-24 (on the midline, 0.5 cun superior to the anterior hairline). |
| Ex-HN-1  (Si shen cong) | A group of four points, each located 1 cun from ➞ GV-20(anterior, posterior and lateral). |
| MS-6  The motor area | 0.5cms posterior to the midpoint of the anterior-posterior line defines the upper limit of the motor area. The lower limit intersects the eyebrow-occiput line at the anterior border of the natural hairline on the temple. |
| MS-7  The sensory area | A line parallel to the motor area and 1.5cms behind it. |
