# Supplementary material for: Additional effects of acupuncture on early comprehensive rehabilitation in patients with mild to moderate acute ischemic stroke: a multicenter randomized controlled trial
Source: BMC Complement Altern Med. 2016 Jul 18;16:226. doi: 10.1186/s12906-016-1193-y (PMC4950630; doi:10.1186/s12906-016-1193-y)
Supplement: Additional file 2: — Ethical approvals of the ethical committees. (PDF 770 kb) [file 12906_2016_1193_MOESM2_ESM.pdf]

Ethical Approvals of the Ethical Committees

The 3rd Affiliated Hospital of Zhejiang Chinese Medical University is held responsible for the project. The trial is being conducted in 3 hospitals (or 3 centers). The protocol has been submitted to the Ethical Committee of the 3rd Affiliated Hospital of Zhejiang Chinese Medical University firstly and passed the ethical review. Then it has been submitted to the ethical committees of 3 hospitals, where the trial will proceed. All the four ethical committees have approved the protocol and the ethical approvals are listed as follows:

1. Ethical approval of the 3rd Affiliated Hospital of Zhejiang Chinese Medical University. (in Chinese)
2. Ethical approval of the 3rd Affiliated Hospital of Zhejiang Chinese Medical University. (in English)
3. Ethical approval of Sir Run Run Shaw Hospital, College of Medicine, Zhejiang University.
4. Ethical approval of the Second Hospital of Jiaxing.
5. Ethical approval of Hangzhou First People's Hospital.

浙江中醫藥大學附屬第三醫院

浙江中医药大学附属第三医院医学伦理委员会

临床试验伦理审批件

试验项目名称	缺血性脑卒中急性期中西医结合康复的临床研究		
研究期限	2012.01.01—2015.12.31	试验类别	多中心随机对照研究
项目负责单位	浙江中医药大学附属第三医院		
合作单位	浙江大学医学院附属邵逸夫医院、嘉兴市第二医院、杭州市第一人民医院		
监督管理部门	浙江省中医药管理局	项目编号	2011ZGG003
医疗机构	浙江中医药大学附属第三医院	主要负责人	姚新苗
申请部门	浙江中医药大学附属第三医院针灸科	临床研究部门	针灸科、神经内科、康复科
项目负责人	方剑乔	职称	教授、主任中医师
批件文号	ZSLL-KY-2012-001-1	批件有效期	2012.01.20-2015.12.31

	审查文件名称	版本	日期
√	伦理审查申请书	1.0	2012.1.15
√	临床研究方案	4.0	2012.1.15
√	知情同意书	2.0	2012.1.15
√	研究者手册	3.0	2012.1.15
√	病例报告表	2.0	2012.1.15

审批意见：

该项目设计科学，研究方法符合人体临床试验伦理学要求，

同意在本单位进行临床试验。

浙江中医药大学附属第三医院
医学伦理委员会（盖章）

2012 年 1 月 20 日

伦理委员会联系电话：0571-88393504
联系地址：浙江省杭州市莫干山路 219 号 （310005）

浙江中醫藥大學附屬第三醫院
Ethics Committee of the 3rd Affiliated Hospital of Zhejiang Chinese Medical University

Ethical Approvals **of Clinical Research**

Project name	Clinical Research on Integrated Rehabilitation with Traditional Chinese and Western Medicine for Acute Stroke		
Commencing and ending date	from July 2011 to June 2015	Type of Research	Multicenter RCT
Charging hospital	The 3rd Affiliated Hospital of Zhejiang Chinese Medical University		
Cooperative hospitals	Sir Run Run Shaw Hospital, College of Medicine, Zhejiang University;<br>The 2nd Hospital of Jiaxing;<br>Hangzhou First People's Hospital		
Supervision and Management department	The Health Bureau of Zhejiang Province	Project number	2011ZGG003
Medical institution	The 3rd Affiliated Hospital of Zhejiang Chinese Medical University	The nerson in charge of the institution	XinMiao Yao
Application department	Department of Acupuncture	Cooperative department	Department of Neurology and Rehabilitation
The person in charge of the project	JianQiao Fang	Title	Professor<br>Deputy President
Approved document	ZSLL-KY-2012-001-1	Period of validity	2012.1-2015.12

	The Contents of Review	Version	Date
√	The application of ethical review	1.0	2012.1.15
√	Study protocol	4.0	2012.1.15
√	Informed consent	2.0	2012.1.15
√	Handbook of researchers	3.0	2012.1.15
√	Case report	2.0	2012.1.15

Opinions of Approval:

The protocol is designed sciencely, and is consistent with human ethical requirements. The ethics committee agreed to conduct the clinical trials.

The 3rd Affiliated Hospital of Zhejiang Chinese Medical University
Ethics Committee
January 20, 2012

TTEL: +86-571-88393504
Address: No.219, Moganshan Road, XiHu District Hangzhou, Zhejiang Province,310005, China.

浙江大学医学院附属
邵逸夫醫院

浙江大学医学院附属邵逸夫医院医学伦理委员会
Ethics Committee of Sir Run Run Shaw Hospital, College of Medicine, Zhejiang University

伦理审查批件

批件文号：科研项目 20120201

项目名称	缺血性脑卒中急性期中西医结合康复的临床研究				
研究期限	2012-01-01 至 2015-12-31			试验类别	多中心随机对照研究
受试者总例数	240	申请医疗机构承担	80	其他机构承担	160
项目来源	浙江省中医药管理局中医药防治重大疾病攻关计划				
监督管理部门	浙江中医药大学附属第三医院			项目编号	2011ZGG003
承担单位	浙江中医药大学附属第三医院			项目负责人	方剑乔
协作单位	浙江大学医学院附属邵逸夫医院<br>嘉兴市第二医院<br>杭州市第一人民医院			分中心负责人	李建华<br>顾旭东<br>陈丽娜

审查内容	审查意见		
立题伦理性	同意☑	修正☐	不同意☐
知情同意书（2.0 版,2012.1.15）	同意☑	修正☐	不同意☐
临床研究方案（4.0 版,2012.1.15）	同意☑	修正☐	不同意☐
病例报告表（2.0 版,2012.1.15）	同意☑	修正☐	不同意☐
研究人员配备	同意☑	修正☐	不同意☐

综合审核意见：

该项目设计科学，研究方法符合人体临床试验伦理学要求，

同意在本单位进行临床试验。

浙江大学医学院附属邵逸夫医院
医学伦理委员会（盖章）
2012 年 2 月 1 日

联系电话：0571-86044817
联系地址：浙江省杭州市庆春东路 3 号

嘉兴市第二医院涉及人体研究的伦理委员会批准书

Ethics Committee Approval on Clinical Research of the 2nd Hospital of Jiaxing

项目名称：浙江省中医药防治重大疾病攻关计划项目-- 中风后遗症中西医结合整体康复的临床研究

项目负责人单位：浙江省针灸推拿医院　　项目负责人：方剑乔 教授

通讯地址：杭州市莫干山路 219 号　　联系方式：0571-87238288

项目分中心研究内容：缺血性脑卒中急性期中西医结合康复治疗的临床研究

项目分中心单位：嘉兴市第二医院

项目分中心负责人姓名学历：顾旭东主任医师　科室：康复医学中心

电话：0573-82073236　　电子邮箱：jxgxd@hotmail.com

通信地址：浙江省嘉兴市环城北路 1518 号康复医学中心

伦理委员会意见：

该项目实验目的明确，实验设计合理，对受试者的选择公正，且受试者承担的风险是最小的，有适当的监督措施，能够确保受试者的安全。并且本项目预期结果意义重大。受试者能够获得书面证明的知情同意。经伦理委员会审查，对该项目的申请予以批准。

伦理委员会主席　[illegible]

日期　2018.3.18

杭州市第一人民医院医学伦理委员会
Ethics Committee of Hangzhou First People's Hospital

伦理审查批件

杭州市第一人民医院伦理委员会 2012 第 006 号

项目名称	缺血性脑卒中急性期中西医结合康复的临床研究				
研究期限	2012-01-01 至 2015-12-31			试验类别	多中心随机对照研究
受试者总例数	240	申请医疗机构承担	80	其他机构承担	160
项目来源	浙江省中医药管理局中医药防治重大疾病攻关计划				
监督管理部门	浙江中医药大学附属第三医院			项 目 编 号	2011ZGG003
承 担 单 位	浙江中医药大学附属第三医院			项 目 负 责 人	方剑乔
协作单位	浙江大学医学院附属邵逸夫医院<br>嘉兴市第二医院<br>杭州市第一人民医院			分中心负责人	李建华<br>顾旭东<br>陈丽娜

审 查 内 容	委 员 投 票 结 果		
立题伦理性	同意☑	修正☐	不同意☐
知情同意书（2.0 版,2012.1.15）	同意☑	修正☐	不同意☐
临床研究方案（4.0 版,2012.1.15）	同意☑	修正☐	不同意☐
病例报告表（2.0 版,2012.1.15）	同意☑	修正☐	不同意☐
研究人员配备	同意☑	修正☐	不同意☐

综合审核意见：

该项目设计科学，研究方法符合人体临床试验伦理学要求，同意在本单位进行临床试验。

杭州市第一人民医院
医学伦理委员会（盖章）
2012年 2 月6日
