# Supplementary material for: Additional effects of acupuncture on early comprehensive rehabilitation in patients with mild to moderate acute ischemic stroke: a multicenter randomized controlled trial
Source: BMC Complement Altern Med. 2016 Jul 18;16:226. doi: 10.1186/s12906-016-1193-y (PMC4950630; doi:10.1186/s12906-016-1193-y)
Supplement: Additional file 5: Table S2. — VFSS scoring criteria. (DOCX 20 kb) [file 12906_2016_1193_MOESM5_ESM.docx]

Additional file 5: Table S2. VFSS scoring criteria.

| Stage | Score | Level |
| --- | --- | --- |
| Oral stage | 0 | Absent oral transit, or transmitting food into the pharyngeal by gravity |
|  | 1 | No bolus, just flowing into the pharyngeal by pieces |
|  | 2 | Delayed oral transit, and incomplete oral clearance after swallow again |
|  | 3 | Complete oral transit |
| Pharyngeal stage | 0 | Insufficient swallow reflex |
|  | 1 | A lot of residue in epiglottic vallecula and pyriform sinu |
|  | 2 | A small amount of residue, and complete oral clearance by swallowing repeatedly |
|  | 3 | Complete pharyngeal transit |
| Aspiration | 0 | Most of the aspiration, but no bucking |
|  | 1 | Most of the aspiration, with bucking |
|  | 2 | A small part of the aspiration, but no bucking |
|  | 3 | A small amount of the aspiration, with bucking |
|  | 4 | No aspiration |
| Total score |  |  |
