# Supplementary material for: Additional effects of acupuncture on early comprehensive rehabilitation in patients with mild to moderate acute ischemic stroke: a multicenter randomized controlled trial
Source: BMC Complement Altern Med. 2016 Jul 18;16:226. doi: 10.1186/s12906-016-1193-y (PMC4950630; doi:10.1186/s12906-016-1193-y)
Supplement: Additional file 6: Table S3. — Summary of adverse reaction of acupuncture. (DOCX 19 kb) [file 12906_2016_1193_MOESM6_ESM.docx]

Additional file 6: Table S3. Summary of adverse reaction of acupuncture.

| Symptom | Group | Mild | | Moderate | | Severe | |  |
| --- | --- | --- | --- | --- | --- | --- | --- | --- |
|  |  | n | % | n | % | n | % | |
| Bleeding | AG | 86 | 47.78 | - | - | - | - | |
| Local hematoma | AG | 25 | 13.89 | - | - | - | - | |
| Unbearable prickling | AG | 12 | 9.60 | - | - | - | - | |
