# Supplementary material for: Additional effects of acupuncture on early comprehensive rehabilitation in patients with mild to moderate acute ischemic stroke: a multicenter randomized controlled trial
Source: BMC Complement Altern Med. 2016 Jul 18;16:226. doi: 10.1186/s12906-016-1193-y (PMC4950630; doi:10.1186/s12906-016-1193-y)
Supplement: Additional file 7: Table S4. — Incidence of adverse events. (DOCX 21 kb) [file 12906_2016_1193_MOESM7_ESM.docx]

Additional file 7: Table S4. Incidence of adverse events.

|  | AG (n=125) | NAG (n=125) |
| --- | --- | --- |
| AEs | 102 | 96 |
| AE relatedness to treatment |  |  |
| Not related | 95 | 88 |
| Possibly/definitely | 2 | 2 |
| Unknown | 5 | 6 |
| Study treatment |  |  |
| Temporarily interrupted | 1 | 2 |
| Permanently discontinued | 1 | 0 |
| SAEs | 7 | 9 |
| SAE criteria |  |  |
| Death | 0 | 0 |
| Life-threatening | 0 | 0 |
| Prolonged hospitalization | 3 | 4 |
| Resulted in disability | 1 | 2 |
| Important medical event | 2 | 1 |
| Other | 1 | 2 |
| SAE relatedness to treatment |  |  |
| Not related | 6 | 7 |
| Possibly/definitely | 0 | 0 |
| Unknown | 1 | 2 |
| Study treatment |  |  |
| Temporarily interrupted | 1 | 2 |
| Permanently discontinued | 2 | 1 |
| Five most common SAEs |  |  |
| Pneumonia | 3 | 4 |
| Urinary tract infection | 2 | 2 |
| Heart failure | 1 | 1 |
| Acute heart disease | 1 | 1 |
| Recurrent stroke | 0 | 1 |
| AE=adverse event, SAE=serious adverse event. | | |
